# Inflammation-Based Scores as a Common Tool for Prognostic Assessment in Heart Failure or Cancer

**DOI:** 10.3389/fcvm.2021.725903

**Published:** 2021-10-22

**Authors:** Henrike Arfsten, Anna Cho, Suriya Prausmüller, Georg Spinka, Johannes Novak, Georg Goliasch, Philipp E. Bartko, Markus Raderer, Heinz Gisslinger, Gabriela Kornek, Wolfgang Köstler, Guido Strunk, Matthias Preusser, Christian Hengstenberg, Martin Hülsmann, Noemi Pavo

**Affiliations:** ^1^Division of Cardiology, Department of Internal Medicine II, Medical University of Vienna, Vienna, Austria; ^2^Division of Oncology, Department of Internal Medicine I, Medical University of Vienna, Vienna, Austria; ^3^Division of Hematology and Hemostaseology, Department of Internal Medicine I, Medical University of Vienna, Vienna, Austria; ^4^Medical Direction, Vienna General Hospital, Medical University of Vienna, Vienna, Austria; ^5^Complexity Research, Vienna, Austria

**Keywords:** prognostic score, HFrEF, oncology, inflammation, nutrition

## Abstract

**Background:** Inflammation-based scores are widely tested in cancer and have been evaluated in cardiovascular diseases including heart failure.

**Objectives:** We investigated the impact of established inflammation-based scores on disease severity and survival in patients with stable heart failure with reduced ejection fraction (HFrEF) paralleling results to an intra-institutional cohort of treatment naïve cancer patients.

**Methods:** HFrEF and cancer patients were prospectively enrolled. The neutrophil-to-lymphocyte-ratio (NLR), the monocyte-to-lymphocyte-ratio (MLR), the platelet-to-lymphocyte-ratio (PLR), and the prognostic nutritional index (PNI) at index day were calculated. Association of scores with disease severity and impact on overall survival was determined. Interaction analysis was performed for the different populations.

**Results:** Between 2011 and 2017, a total of 818 patients (443 HFrEF and 375 cancer patients) were enrolled. In HFrEF, there was a strong association between all scores and disease severity reflected by NT-proBNP and NYHA class (*p* ≤ 0.001 for all). In oncologic patients, association with tumor stage was significant for the PNI only (*p* = 0.035). In both disease entities, all scores were associated with all-cause mortality (*p* ≤ 0.014 for all scores). Kaplan–Meier analysis confirmed the discriminatory power of all scores in the HFrEF and the oncologic study population, respectively (log-rank *p* ≤ 0.026 for all scores). A significant interaction with disease (HFrEF vs. cancer) was observed for PNI (*p*_interaction_ = 0.013) or PLR (*p*_interaction_ = 0.005), respectively, with higher increase in risk per inflammatory score increment for HFrEF.

**Conclusion:** In crude models, the inflammatory scores NLR, MLR, PLR, and PNI are associated with severity of disease in HFrEF and with survival in HFrEF similarly to cancer patients. For PNI and PLR, the association with increase in risk per increment was even stronger in HFrEF than in malignant disease.

## Introduction

Heart failure with reduced ejection fraction (HFrEF) is a major cause of morbidity and mortality in developed countries (1). Comparable to cancer, previous research emerges that show that elevated inflammatory markers are characteristic in patients with HFrEF and correlate with disease severity and prognosis (2, 3). This is reflected by a steady immune activation and low-grade inflammation in response to various stimuli including hypoxia, proinflammatory cytokines, and neurohormonal activation (4, 5). Sustained expression and exposure to cytokines and proteolytic enzymes can lead to myocardial remodeling and altered cardiac metabolism resulting in ventricular remodeling and dysfunction, negative inotropic effects, and heart failure (HF) progression (6, 7). Especially in more advanced stages of HF, the expression of inflammatory mediators leads to detrimental effects on other systems contributing to more systemic deterioration and end organ damage in the course of the chronic disease (8, 9).

In recent years, the role of this low-grade systemic inflammatory activity in chronic diseases generally and especially in cancer development and progression has been investigated more thoroughly, proving an association between the level of chronic inflammation and worse outcome (7, 10, 11). Consequently, inflammation-based prognostic scores have been introduced as a simple index of systemic inflammation and suggested for improved risk stratification. The neutrophil-to-lymphocyte ratio (NLR), the monocyte-to-lymphocyte ratio (MLR), and the platelet-to-lymphocyte ratio (PLR), as well as the prognostic nutritional index (PNI) share mutual quantitative relationships of blood cells and inflammatory activity. Inflammation-based scores are widely tested in cancer (12–28) and have been evaluated in primarily acute settings of cardiovascular diseases including heart failure (29–37). We aimed to broaden knowledge by investigating the impact of established inflammation-based scores on disease severity and survival in patients with stable chronic HFrEF and, more important, paralleling results to an intra-institutional cohort of treatment naïve cancer patients.

## Methods

The study protocol complies with the Declaration of Helsinki and was approved by the ethics committee of the Medical University of Vienna. All included patients had to be at least 18 years of age and provided written informed consent to study participation.

### Study Population

Study participants for this observational, non-interventional study were recruited at the heart failure and the oncologic outpatient clinic at the Vienna General Hospital, both university-affiliated tertiary centers, respectively. Patients with stable chronic HFrEF undergoing routine ambulatory care have been identified from a prospective registry. Heart failure with reduced ejection fraction was defined in line with the guidelines as a history of HF signs and symptoms as well as a documented left ventricular ejection fraction below 40% (1). Baseline examination included medical history, detailed assessment of current medication, and an electrocardiogram. Cardiovascular risk factors were recorded (1). Routine laboratory parameters for the respective visit were available.

Second, consecutive patients with a primary diagnosis of cancer but treatment naïve were prospectively enrolled. To avoid inflammatory bias, eligible patients were excluded if they showed clinical signs of infection. Patients were classified according to tumor entity and tumor stage. Comorbidities and current medical therapy were recorded. Further details on the patient cohort have been published previously (38–41). The present analysis incorporates a subpopulation of patients for whom data for the calculation of respective risk scores were available.

All-cause mortality was defined as the primary endpoint and information on death was obtained from the Austrian Central Office of Civil Registration.

### Laboratory Assessment

Laboratory parameters were assessed from venous blood samples drawn from a peripheral vein at the respective outpatient clinic visit. Routinely available laboratory parameters were analyzed according to local laboratory standard procedures.

### Assessment of Risk Scores

All scores were assessed as previously defined (12, 14, 18, 25). The NLR, MLR, PLR, and PNI were defined as the ratios of the measured parameters (absolute numbers) as follows:

NLR: the ratio of neutrophils (g/L) and lymphocytes (g/L),
MLR: the ratio of monocytes (g/L) and lymphocytes (g/L),
PLR: ratio of platelets (g/L) and lymphocytes (g/L),
PNI: product of albumin and the total lymphocyte count [PNI = albumin (g/L) × total lymphocyte count (g/L)].

### Statistical Analysis

Continuous variables are presented as median and interquartile range (IQR) and compared by using the Mann–Whitney *U*-test. Discrete data were presented as count and percentage and analyzed by using a χ^2^ test. Central tendencies and correlation between assessed risk scores and disease severity reflected by New York Heart Association (NYHA) functional class and N-terminal pro-brain natriuretic peptide (NT-proBNP) (HFrEF population), or tumor-stage (oncologic population; solid tumors only), were compared by Kruskal–Wallis test and Spearman-Rho correlation coefficient, respectively. For outcome analysis, univariate and multivariate Cox proportional hazard regression analysis was applied to estimate hazard ratios (HRs) with 95% confidence interval (CI). Interaction analysis was performed to test differences in risk prediction between HFrEF and cancer of the respective scores. Kaplan–Meier analysis was applied to illustrate the prognostic ability of the respective scores for strata of tertiles whereas groups were compared using the log-rank test. Two-sided *p* < 0.05 were used to indicate statistical significance. The SPSS 24.0 software (IBM Corp, New York, NY) was used for all analyses. Illustrations were created with GraphPad Prism version 9.1.0.

## Results

### Baseline Characteristics

#### Overall Study Population

Baseline characteristics of the study patients are detailed in Table 1. The median age of 818 enrolled patients was 63 years (IQR: 53–72 years) and 474 (58%) of the patients were men. The body mass index of the cohort was 26.1 kg/m^2^ (IQR: 23.0–29.7). Median hematologic blood parameters hemoglobin [13.4 g/dl (IQR: 12.1–14.4)], leukocytes [235 g/L (IQR: 186–285)], platelets [7.41 g/L (IQR: 6.01–9.29)], neutrophils [5.4 g/L (IQR: 3.9–7.0)], monocytes [0.6 g/L (IQR: 0.5–0.8)], and lymphocytes [1.4 g/L (IQR: 1.1–1.9)] ranged within the normal reference values of the institution's standard laboratory. The two study cohorts HFrEF vs. oncologic patients are presented in the following. Among others, both study cohorts showed significant differences in blood pressure (*p* < 0.001), comorbidities [coronary artery disease (*p* < 0.001), diabetes (*p* < 0.001), and atrial fibrillation (*p* < 0.001)], kidney function (creatinine; *p* < 0.001), and NT-proBNP (*p* < 0.001), which is however in line with the respective disease entity. More details on baseline characteristics of both study cohorts are presented in Table 1.

**Table 1:** Baseline characteristics of the two study populations.

	**Overall study population (*n*= 818)**	**HFrEF study population (*n* = 443)**	**Oncologic study population (*n* = 375)**	***p*-value**
Age, median years (IQR)	63 (53–72)	64 (53–72)	62 (53–71)	0.299
Male sex, *n* (%)	474 (58)	325 (73)	149 (40)	<0.001
BMI, kg/m^2^ (IQR)	26.1 (23.0–29.7)	26.6 (23.8–30.2)	25.2 (22.6–29.0)	<0.001
Systolic BP, mmHg (IQR)	133 (120–150)	130 (114–146)	138 (125–150)	<0.001
Diastolic BP, mmHg (IQR)	80 (74–90)	80 (70–89)	85 (78–91)	<0.001
Heart rate, bpm (IQR)	71 (633–81)	71 (62–80)	73 (65–83)	0.042
**Comorbidities**				
CAD, *n* (%)	230 (28)	208 (47)	22 (6)	<0.001
Diabetes mellitus, *n* (%)	166 (20)	134 (30)	32 (9)	<0.001
Arterial hypertension, *n* (%)	325 (40)	158 (36)	167 (45)	0.051
Atrial fibrillation, *n* (%)	115 (14)	103 (23)	12 (3)	<0.001
**NYHA functional class**				
NYHA I, *n* (%)		68 (15)	–	–
NYHA II, *n* (%)		178 (40)	–	–
NYHA III, *n* (%)		164 (37)	–	–
NYHA IV, *n* (%)		9 (2)	–	–
Cancer disease stage^*^			***n*** = 348^*^	
Stage I, *n* (%)	–	–	64 (18)	–
Stage II, *n* (%)	–	–	41 (12)	–
Stage III, *n* (%)	–	–	94 (27)	–
Stage IV, *n* (%)	–	–	149 (43)	–
**Laboratory parameters**				
Hemoglobin, g/dl (IQR)	13.4 (12.1–14.4)	13.3 (12.1–14.6)	13.4 (12.1–14.3)	0.611
Platelet count, g/L (IQR)	235 (186–285)	225 (178–261)	258 (203–305)	<0.001
Leucocytes, g/L (IQR)	7.4 (6.1–9.3)	8.1 (6.3–9.1)	7.2 (5.7–9.6)	0.243
Neutrophils, g/L (IQR)	5.4 (3.9–7.0)	5.8 (4.6–7.3)	4.6 (3.3–6.4)	<0.001
Monocytes, g/L (IQR)	0.6 (0.5–0.8)	0.7 (0.6–0.9)	0.5 (0.4–0.7)	<0.001
Lymphocytes, g/L (IQR)	1.4 (1.1–1.9)	1.5 (1.1–2.0)	1.4 (1.0–1.8)	0.003
Sodium, mmol/L (IQR)	139 (138–141)	140 (138–142)	139 (137–141)	0.003
Potassium, mmol/L (IQR)	4.5 (4.2–4.9)	4.8 (4.1–5.1)	4.3 (4.0–4.5)	<0.001
Bilirubin, mg/dl (IQR)	0.6 (0.4–0.8)	0.7 (0.4–0.9)	0.6 (0.4–0.7)	0.277
Cholinesterase, kU/L (IQR)	7.1 (5.8–8.3)	6.10 (5.6–8.3)	7.2 (6.0–8.3)	0.119
Gamma-GT, U/L (IQR)	40 (24–88)	50 (27–105)	33 (22–67)	<0.001
LDH, U/L (IQR)	194 (167–230)	213 (174–230)	187 (162–230)	0.015
AST, U/L (IQR)	24 (19–31)	28 (20–30)	23 (18–31)	0.058
ALT, U/L (IQR)	22 (16–32)	28 (17–32)	21 (16–32)	0.146
Total cholesterol, mg/dl (IQR)	185 (152–223)	171 (138–201)	209 (175–238)	<0.001
Triglycerides, mg/dl (IQR)	117 (86–164)	114 (84–157)	121 (91–191)	0.008
Albumin, g/L (IQR)	43.0 (39.9–45.5)	43.3 (40.3–45.7)	42.5 (39.4–44.9)	0.002
Creatinine, mg/dl (IQR)	1.0 (0.8–1.3)	1.4 (0.10–1.6)	0.9 (0.8–1.0)	<0.001
BUN, mg/dl (IQR)	18 (13–26)	28(16–33)	15 (12–19)	<0.001
CK, U/l (IQR)	77 (52–111)	80 (57–117)	71 (46–101)	<0.001
NT-proBNP, pg/ml (IQR)	545 (131–2,361)	2,053 (842–4,345)	133 (70–297)	<0.001
**Heart failure medication**				
RAS blockade, *n* (%)	525 (64)	414 (94)	111 (30)	<0.001
Beta-Blockers, *n* (%)	495 (61)	413 (93)	82 (22)	<0.001
MRA, *n* (%)	328 (40)	318 (72)	10 (3)	<0.001

*Values are median [interquartile range (IQR)] or n (%). ALT, alanine aminotransferase; AST, aspartate aminotransferase; BMI, body mass index; BP, blood pressure; bpm, beats per minute; BUN, blood urea nitrogen; CAD, coronary artery disease; CK, creatinine kinase; IQR, interquartile range; LDH, lactate dehydrogenase; MRA, mineralocorticoid antagonist; NT-proBNP, N-terminal pro B-type natriuretic peptide; NYHA, New York Heart Association; RAS, renin–angiotensin system*.

* *In 348/379 patients, tumor stage was assessed by the respective treating oncologist and was indicated for all patients excluding those with myeloproliferative neoplasms*.

#### HFrEF Study Population

A total of 443 stable chronic HFrEF patients were enrolled between 2011 and 2017. Most patients presented in NYHA functional class II and III [178 (40%) and 173 (39%), respectively], with a median NT-proBNP of 2,053 pg/ml (IQR: 842–4,345). Guideline recommended heart failure therapy was well-established with 413 (93.2%) patients on betablocker, 414 (93.5%) patients receiving renin–angiotensin system inhibitors (RASi) [angiotensin converting enzyme inhibitor (ACEi), angiotensin receptor blocker (ARB), or angiotensin receptor-neprilysin inhibitor (ARNi)], and 318 (71.8%) patients were on a mineralocorticoid-receptor antagonist (MRA).

#### Oncologic Study Population

The oncologic study population was composed of a total of 375 treatment-naïve cancer patients enrolled between 2011 and 2013. Most of them suffered from breast cancer [109 (29%)], followed by lung cancer [61 (16%)], gastrointestinal tumors [52 (14%)], or myelodysplastic malignancies [47 (13%)]. A detailed overview on tumor entity is listed in Supplementary Table 1. Among the patients with solid tumors (*n* = 348), most presented with more advanced tumor stage III and IV [94 (27%) and 149 (43%), respectively].

### Association Between Risk Scores and Disease Severity

All scores differed significantly between both disease entities (Table 2).

**Table 2:** Prognostic scores in HFrEF vs. cancer.

**Prognostic scores/ratios**	**HFrEF study population** **(*n* = 443)**	**Oncologic study population** **(*n* = 375)**	***p*-value**
NLR, –(IQR)	3.8 (2.6–5.7)	3.3 (2.1–5.4)	0.001
MLR, –(IQR)	0.5 (0.4–0.7)	0.4 (0.3–0.6)	<0.001
PLR, –(IQR)	145 (110–202)	179 (129–269)	<0.001
PNI, –(IQR)	65 (46–86)	58 (40–81)	0.002

*MLR, monocyte-to-lymphocyte ratio; NRI, nutritional risk index; NLR, neutrophil-to-lymphocyte ratio; PLR, platelet-to-lymphocyte ratio, PNI, prognostic nutritional index*.

**Figure 1:**
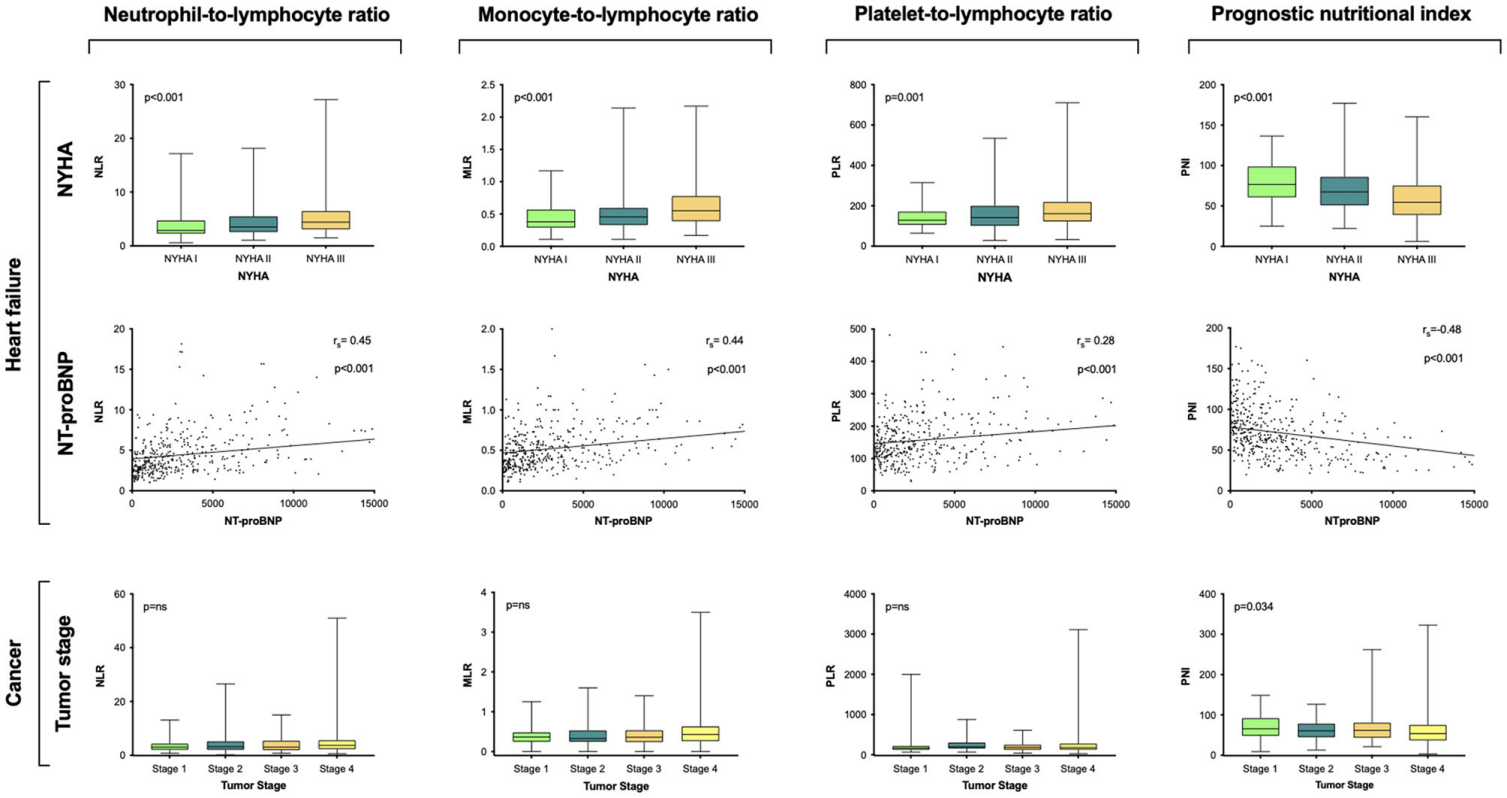
Association of assessed risk scores with disease severity. Group comparisons between respective risk score with NT-proBNP, NYHA classification, and tumor stage were calculated by Spearman-Rho correlation and Kruskal–Wallis test, respectively. Levels are displayed as Tukey boxplots. Scatter plots depict the relation between NT-proBNP and assessed scores NLR, MLR, PLR, and PNI. NT-proBNP, N-terminal pro B-type natriuretic peptide; NYHA, New York Heart Association; NLR, neutrophil-to-lymphocyte ratio; MLR, monocyte-to-lymphocyte ratio; PLR, platelet-to-lymphocyte ratio; PNI, prognostic nutritional index.

In the HFrEF study cohort, all scores were associated with disease severity reflected by NYHA classification (NLR *p* < 0.001; MLR *p* < 0.001; PLR *p* = 0.001; PNI *p* < 0.001) and NT-proBNP (NLR rs = 0.45, *p* < 0.001; MLR rs = 0.44, *p* < 0.001; PLR rs = 0.28 *p* = 0.001; PNI rs = −0.48, *p* < 0.001; Figure 1). For assessment of association between respective risk score and disease severity in cancer patients reflected by tumor stages, risk scores were analyzed in the respective study subpopulation with solid tumors only (*n* = 348). Solely values for the PNI reached statistical significance (*p* = 0.034; Figure 1).

### Predictive Power of Assessed Risk Scores and Outcome

**Figure 2:**
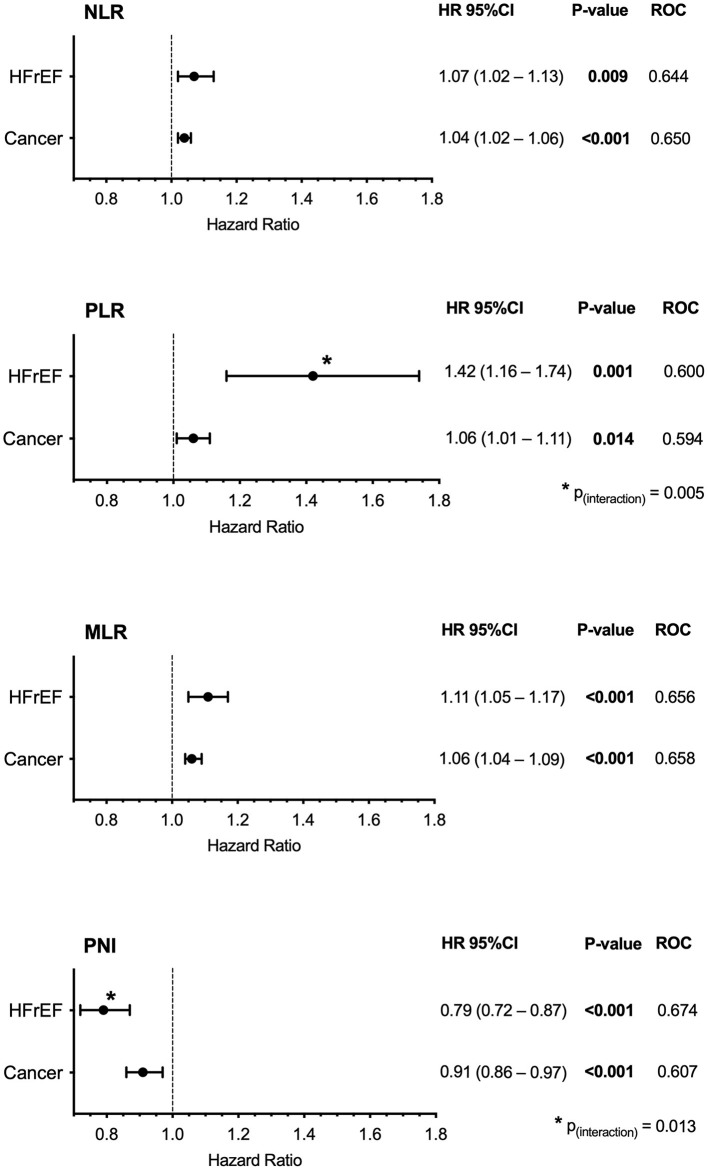
Association of inflammatory scores with outcome in stable HFrEF (*n* = 443) or treatment naïve cancer (solid and hematologic cancer) (*n* = 375). Cox regression and ROC analysis of different inflammation-based prognostic scores are shown. NLR, Neutrophil-to-lymphocyte ratio; MLR, Monocyte-to-lymphocyte ratio; PLR, Platelet-to-lymphocyte ratio; PNI, Prognostic nutritional index.

**Figure 3:**
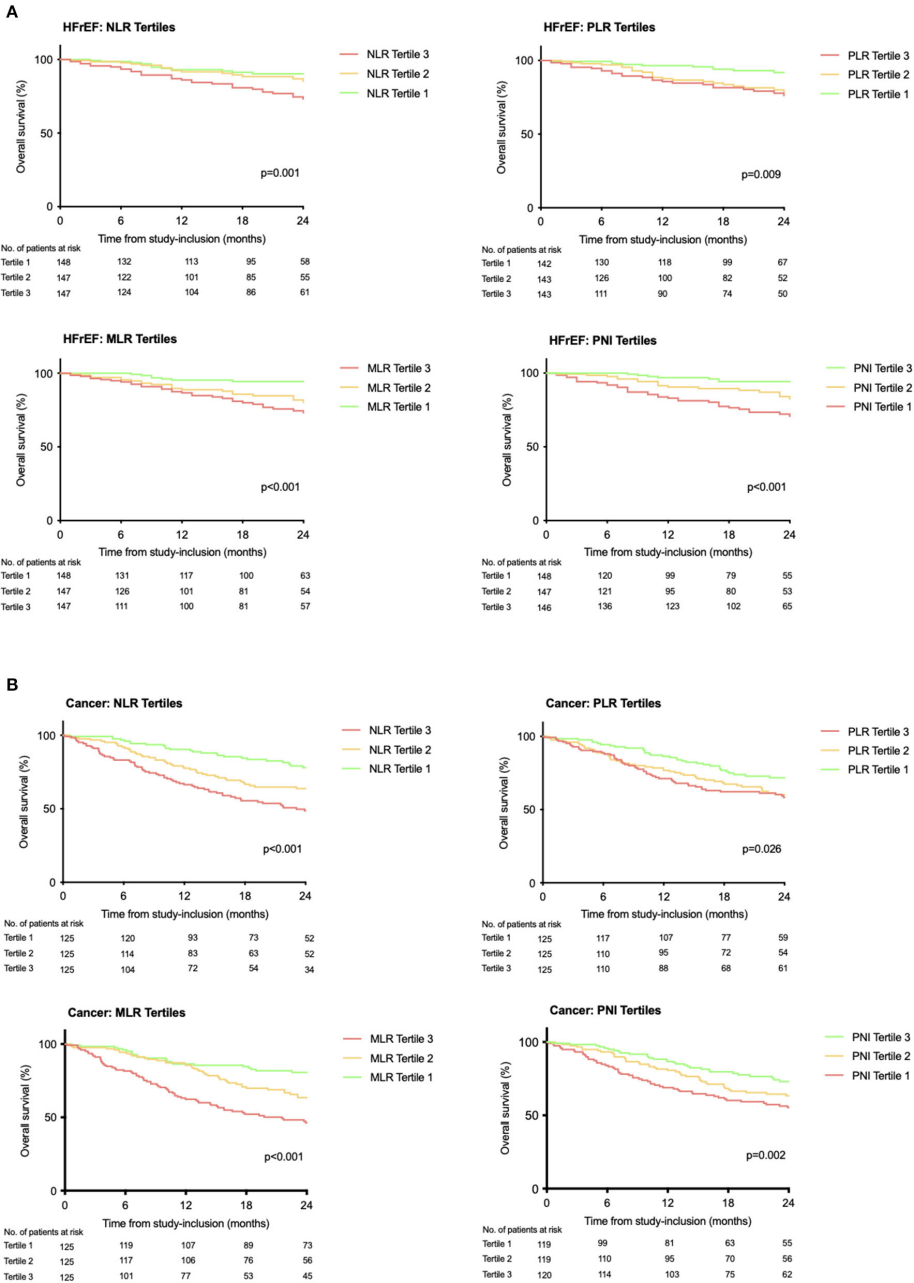
Kaplan–Meier estimates for overall survival in stable HFrEF **(A)** or treatment naïve cancer **(B)** according to prognostic scores. Assessed for within-population tertile strata. Curves were compared by the log-rank test.

During a median follow-up of 22 months (IQR: 11–30), 231 (28%) patients died [HFrEF: follow-up: median 21 months (IQR 10–28), *n* = 75 (17%); cancer: follow-up: median 22 months (IQR 14–33), *n* = 156 (41%); *p* < 0.001 (Supplementary Figure 1)]. All assessed risk scores were associated with all-cause mortality in crude Cox regression analysis in HFrEF or in cancer, respectively (*p* ≤ 0.014 for all, Figure 2). In the HFrEF study population, except for the NLR, all scores remained significantly associated with outcome after adjustment for NYHA classification, physical confounders reflected by age, sex, and BMI, or for laboratory parameters. The PNI remained significantly associated with outcome even after adjustment for NT-proBNP, the most prominent heart failure specific biomarker (Supplementary Table 2A). In the cancer study population, all scores remained significantly associated with outcome after adjustment for tumor stage (in the solid tumor study population only). Solely, the PLR lost significance when adjusting for the physical status confounder model and the laboratory confounder model (Supplementary Table 2B). Interaction analysis revealed significance between disease (HFrEF vs. cancer) and the PNI or the PLR, respectively, with higher increase in risk per inflammatory score increment for HFrEF (PNI: *p*_interaction_ = 0.013; PLR: *p*_interaction_ = 0.005). This was not observed for the NLR (*p*_interaction_ = 0.258) or the MLR (*p*_interaction_ = 0.192). The Kaplan–Meier analysis confirmed the discriminatory power of the scores for strata of tertiles for both cohorts (log-rank HFrEF study population: NLR *p* = 0.001, PLR *p* = 0.009, MLR *p* < 0.001, PNI *p* < 0.001; log-rank oncology study population: NLR *p* < 0.001, PLR *p* = 0.026, MLR *p* < 0.001. PNI *p* = 0.002; Figure 3).

## Discussion

The present long-term observational study showed inflammation-based risk scores NLR, MLR, PLR, and PNI, originally developed for prognostic assessment in malignancies, as a potent tool for refining risk in stable chronic HFrEF. The analyses directly contrasted the ability of the scores to predict outcome in patients with malignancies or with heart failure. We found that (i) in the HFrEF study population, the association with disease severity was significant for all scores; (ii) all scores were significantly associated with outcome at long-term follow-up in both disease entities, HFrEF or cancer, with ROC highest for NLR and PNI; and (iii) while PLR and PNI show a significant interaction with respect to the underlying disease, NLR and MLR do not.

### Shared Pathophysiologic Inflammatory Pathways Reflected by Ratios of Inflammatory Cell Lines in HFrEF and Cancer

Besides other joint risk factors (42), also the involvement of systemic inflammation is a recognized crucial pathophysiologic factor in disease development and progression, which is shared between HF and cancer. Several studies provided evidence of enhanced inflammation in either HF or cancer (3). Indeed, in heart failure, disease progression has been attributed to, among others, general but subclinical and non-specific inflammatory processes that have been associated with outcome (9, 11, 43). Nevertheless, the distinct immunologic mechanism is not entirely understood, but several players have been recognized. The specific dysregulated cell lines of interest include neutrophils, monocytes, platelets, and leucocytes (44, 45). All of these cells are responsible for inflammatory event cascades. They induce and control specific programs involving complex series of paracrine and endocrine signals, attract additional cells, stimulate the production and secretion of pro-inflammatory factors, and induce the expression of receptors or factors that are receptive to additional inflammatory signals potentially contributing to disease progression (45). Comparable activated mechanisms of the immune system have been implied in the development and progression of malignancies (45). With the observation that systemic inflammation features circulating white blood cells' and platelets' alteration, the calculation of blood cell-based ratios/scores, as also examined in our study, was established. Thereby, it is sought to assess and further understand the burden and respective prognostic significance of subclinical inflammatory disarrangements. The presented data of the two distinct populations of heart failure and cancer patients may improve our understanding about shared pathways between the two diseases thereby adding to the field of cardio-oncology.

Since the respective score calculation was primarily described for oncological patients, a wide range of analyses can be found in the literature. These include cancer entity-specific evaluations in, among others, lung cancer (12–14), head and neck malignancies (15, 16), gynecologic (17) and gastrointestinal cancers entities (18–20), and hematologic malignancies (21, 22), but also prediction analyses of scores independent of the tumor site of origin (23). The individual expressions of the ratios vary between the respective study cohorts, making it difficult to define uniform cutoff values. However, the respective trends of each score are consistent and comparable with those of our study population. Thus, evidence from our and previous analyses indicates that increased NLR, PLR, or MLR are each of significant prognostic value for outcome assessment in cancer. For the first time, the prognostic value was also shown for patients without prior anticancer therapy, demonstrating that the inflammatory activation can be attributed to the underlying malignant disease.

To date, only few studies addressed the use of the aforementioned scores in cardiovascular outcome assessment. Data available show association between PLR and 30-day mortality in acute heart failure (33). In these patients, the NLR was shown to be associated with 30-day mortality, readmission rate, and long-term outcome (33, 34). In addition, the score reveals association with mortality or heart transplantation in advanced heart failure (35). The risk assessment for heart failure development by MLR has been described for patients undergoing coronary angiography. In these patients, MLR was related to heart failure markers NT-proBNP, left ventricular ejection fraction, and HF hospitalizations during follow-up (36). We extended existing data with our analysis by showing prognostic validity of the scores in stable chronic HFrEF including a long-term follow-up time period. The data revealed a significant association of NLR, PLR, and MLR with disease severity, reflected by NT-proBNP and NYHA functional class, and outcome. More important, in direct comparison to cancer patients, an equipotent relevance of these markers is shown for both diseases.

### The Prognostic Nutritional Index, a Rather Inflammatory Score

Even though serum albumin levels are widely used to determine the patient's nutritional status, in heart failure, hypoalbuminemia has been found present to a similar extent in lean, overweight, and obese patients (46). Furthermore, the positive overall correlation of hypoalbuminemia and inflammation with prognosis in patients with certain chronic diseases, such as cancer (47), heart failure (46), and end-stage renal disease (48), and both factors' mutual association (49) indicate inflammation to be among the underlying etiologies of hypoalbuminemia.

The PNI, calculated by lymphocytes and albumin, is often interpreted as a pure nutritional marker. However, the score, based on parameters representing driving forces in inflammatory processes, should be interpreted equivalently as a reflection of systemic inflammation in the course of diseases (25). In our study populations, PNI was negatively correlated with outcome and disease severity, respectively. This observation is supported by previous data in cardiovascular disease or cancer (50). Initially, the score has been designed for gastro-intestinal malignancies (19, 24, 25). Since then, its applicability has been investigated in the extended oncological field, including, e.g., ovarian (26), lung (27), or renal cancer (28). With regard to cardiology, there are data on myocardial infarction, chronic, and acute heart failure that support the applicability of PNI for prognostic assessment of these patients (30–32, 35).

## Limitations

The study reflects the experience of a single tertiary care center only. However, this ensures the inclusion of a homogenous patient population with adherence to a consistent clinical routine. Moreover, the study design allows for the so far unique, interdisciplinary direct comparison between two disease entities, which also enables interaction analyses between disease pattern and respective risk score.

## Conclusion

In crude models, the inflammatory scores NLR, MLR, PLR, and PNI are associated with severity of disease in heart failure and with survival HFrEF similar to cancer patients. For PNI and PLR, the association with greater increase in risk per increment was even stronger in HFrEF than in malignant disease, whereas there was no disease-dependent interaction in NLR and MLR.

## Data Availability Statement

The dataset is not intended for forwarding to third parties. Requests for access to specific questions should be addressed to the corresponding author.

## Ethics Statement

This study involving human participants was reviewed and approved by Ethics Committee of the Medical University of Vienna. The patients/participants provided their written informed consent to participate in this study.

## Author Contributions

HA, NP, and MH designed the experiment and wrote the manuscript. HA, NP, AC, SP, GSp, JN, GG, PB, MR, HG, GK, and WK managed participant recruitment and were responsible for sample acquisition. HA, NP, MH, GG, PB, and GSt analyzed and interpreted the data. MH was the supervisor of the study. All authors contributed to manuscript revision, gave important intellectual content, read, and approved the submitted version.

## Funding

The work was supported by a grant from the Austrian Science Fund (KLI 700-B30).

## Conflict of Interest

The authors declare that the research was conducted in the absence of any commercial or financial relationships that could be construed as a potential conflict of interest.

## Publisher's Note

All claims expressed in this article are solely those of the authors and do not necessarily represent those of their affiliated organizations, or those of the publisher, the editors and the reviewers. Any product that may be evaluated in this article, or claim that may be made by its manufacturer, is not guaranteed or endorsed by the publisher.
