# Supplementary material for: Inflammation-Based Scores as a Common Tool for Prognostic Assessment in Heart Failure or Cancer
Source: Front Cardiovasc Med. 2021 Oct 22;8:725903. doi: 10.3389/fcvm.2021.725903 (PMC8569110; doi:10.3389/fcvm.2021.725903)
Supplement: Supplementary file 1 [file Table_1.pdf]

Supplementary Material

1 Supplementary Table S1. Tumor entities of treatment-naïve cancer patients (n=375)

Counts are given as numbers and percentages.

	**Cancer patients (n=375)**
**Tumor entity**	
Breast cancer, n (%)	109 (29.1)
Lung cancer, n (%)	61 (16.3)
Gastrointestinal tumors, n (%)	52 (13.9)
Myelodysplastic malignancies, n (%)	47 (12.5)
ENT-tumor, n (%)	32 (8.5)
Brain tumor, n (%)	22 (5.9)
Myeloproliferative neoplasia, n (%)	12 (3.2)
Esophageal cancer, n (%)	9 (2.4)
Sarcoma, n (%)	7 (1.9)
Neuroendocrine tumor, n (%)	4 (1.1)
Renal cell carcinoma, n (%)	4 (1.1)
Mesothelioma, n (%)	3 (0.8)
Prostate cancer, n (%)	2 (0.5)
Skin cancer, n (%)	2 (0.5)
Urogenital tumors, n (%)	2 (0.5)
Thymoma, n (%)	1 (0.3)
Other, n (%)	6 (1.6)

ENT – ear, nose, throat; Myelodysplastic malignancies – hematological malignancies with abnormal differentiation of myeloid or lymphoid cell lines (e.g. AML, ALL, lymphomas, multiple myeloma); Myeloproliferative neoplasia – hematologic neoplasia with normal cell differentiation (e.g. essential thrombocytosis, polycythemia vera, myelofibrosis).
