# Supplementary material for: Inflammation-Based Scores as a Common Tool for Prognostic Assessment in Heart Failure or Cancer
Source: Front Cardiovasc Med. 2021 Oct 22;8:725903. doi: 10.3389/fcvm.2021.725903 (PMC8569110; doi:10.3389/fcvm.2021.725903)
Supplement: Supplementary file 2 [file Table_2.pdf]

Supplementary Material

Supplementary Table S2. Association of inflammatory scores with outcome in stable HFrEF and treatment naïve cancer patients.

2A. HFrEF (n=443)

Score	Adj.HR[a)]	p-value	Adj.HR[b)]	p-value	Adj.HR[c)]	p-value	Adj HR[d)]	p-value
NLR	1.03 (0.95 – 1.11)	0.514	1.04 (0.98-1.11)	0.166	1.04 (0.98 – 1.03)	0.177	1.05 (0.99-1.12)	0.088
MLR	1.21 (1.11 – 1.32)	0.075	1.08 (1.01-1.15)	**0.017**	1.07 (1.01 – 1.15)	**0.033**	1.10 (1.03-1.17)	**0.006**
PLR	1.07 (0.99 – 1.15)	0.306	1.31 (1.04-1.66)	**0.024**	1.31 (1.05 – 1.62)	**0.015**	1.26 (1.00-1.004)	**0.047**
PNI	0.89 (0.80 – 0.98)	**0.017**	0.83 (0.75-0.91)	**<0.001**	0.82 (0.73 – 0.91)	**<0.001**	0.84 (0.76-0.92)	**<0.001**

2B. Cancer (n=375)

Score	Adj.HR[e)]	p-value	Adj.HR[c)]	p-value	Adj HR[d)]	p-value
NLR	1.03 (1.01 – 1.05)	**0.007**	1.03 (1.00 – 1.05)	**0.025**	1.03 (1.01-1.06)	**0.010**
MLR	1.04 (1.01 – 1.07)	**0.004**	1.05 (1.01 – 1.08)	**0.005**	1.06 (1.03-1.10)	**<0.001**
PLR	1.06 (1.03 – 1.12)	**0.013**	1.05 (0.99 – 1.11)	0.058	1.01 (0.99-1.09)	0.156
PNI	0.94 (0.89 – 0.99)	**0.024**	0.94 (0.89 – 0.99)	**0.030**	0.94 (0.89-0.99)	**0.028**

a) NT-proBNP
b) NYHA
c) Physical status confounder model: Sex, age, BMI
d) Laboratory confounder model: creatinine, LDH, gamma-GT
e) Tumor stage (n=348; solid tumors only)
