# Supplementary material for: Inflammation-Based Scores as a Common Tool for Prognostic Assessment in Heart Failure or Cancer
Source: Front Cardiovasc Med. 2021 Oct 22;8:725903. doi: 10.3389/fcvm.2021.725903 (PMC8569110; doi:10.3389/fcvm.2021.725903)
Supplement: Supplementary file 3 [file Image_1.pdf]

Supplementary Material

Supplemental Figure S1. Kaplan-Meier estimates for overall survival according to the disease entity heart failure or treatment naïve cancer. Curves were compared by the log-rank test.

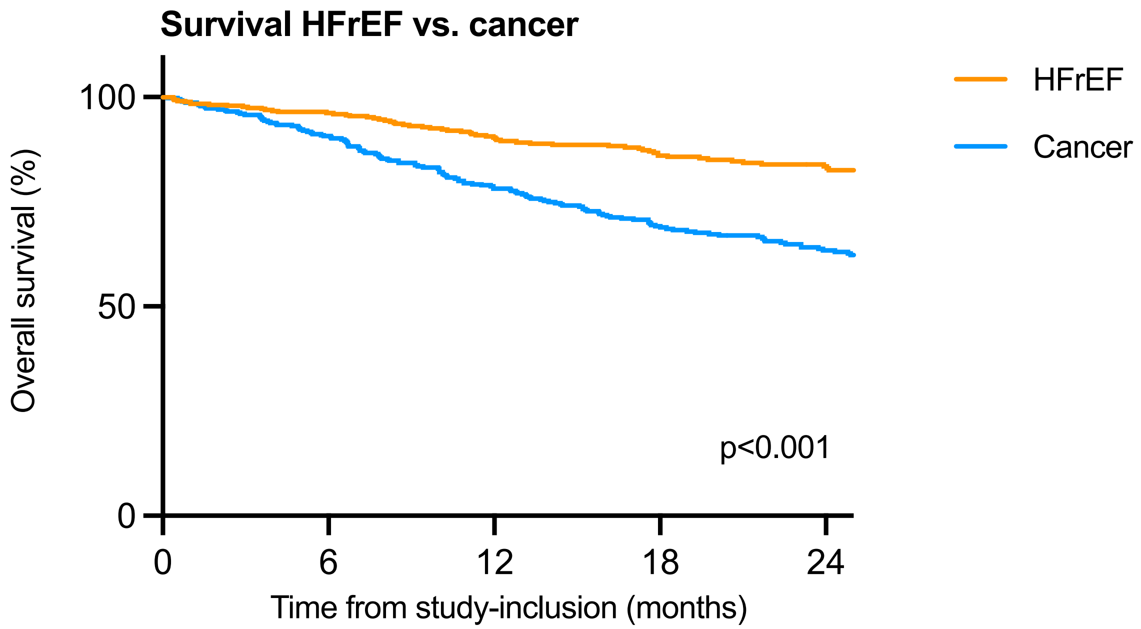

No. of patients at risk

HFrEF	443	381	320	264	176
Cancer	375	339	293	220	176
